# Assessing the role of Computed Tomography Pulmonary Angiogram in diagnosing right heart strain related to acute pulmonary embolism

**DOI:** 10.2174/0118743064508829261003170120

**Published:** 2026-10-09

**Authors:** Sean Landers, James O’Regan, Roisin Rynne, Michael M. Maher, Ronan Curtin, Deirdre Doyle, Desmond M. Murphy

**Affiliations:** 1 Department of Respiratory Medicine, Cork University Hospital, Cork, Ireland; 2 Department of Radiology, Cork University Hospital, Cork, Ireland; 3 Department of Cardiology, Cork University Hospital, Cork, Ireland

**Keywords:** Pulmonary embolism, Right heart strain, CTPA, Echocardiogram, Troponin, BNP

## Abstract

**Introduction/Objective:**

Right heart strain (RHS) is a commonly occurring and significant complication of acute pulmonary embolism (PE). CTPA images often suggest pulmonary hypertension; however, diagnostic echocardiography is not always immediately available to confirm this. In this study, we assessed how accurately Computed Tomography Pulmonary Angiography (CTPA), troponin, and NT-proBNP predict evidence of RHS in patients with acute PE, compared with transthoracic echocardiography (TTE).

**Methods:**

A total of 656 patients had CTPAs performed over 6 months at a tertiary university hospital. Studies positive for PE were then separated into those with and without reported CTPA evidence of RHS. Patients’ TTE, troponin-T, NT-proBNP, and D-dimer results were assessed, and potential relationships were explored.

**Results:**

About 128 (19.5%) studies were positive for acute PE, and 25 patients with RHS on CTPA had TTEs, with 16 showing evidence of RHS. A total of 39 patients with PEs and no CTPA-reported RHS had TTEs, with 7 suggesting RHS. CTPAs showed a 69.6% sensitivity and 78.0% specificity in predicting RHS. NT-pro-BNP (cut-off 300pg/ml) and troponin-T (cut-off 34ng/L) showed higher sensitivity, 92.9% and 75%, respectively, and lower specificity, 33.3% and 58%, respectively, than CTPA. 329/656 (50%) had an alternative explanation for symptoms on CTPA, meaning only 30.5% of patients had no explanation for symptoms.

**Discussion:**

While an accessible and useful test, CTPA is not as reliable in predicting RHS in patients with acute PE as TTE.

**Conclusion:**

Patients diagnosed with a clinically significant PE should be considered for TTE regardless of whether or not RHS is evident on CTPA.

## 1 INTRODUCTION

Right ventricular failure (RVF) is a well-documented and serious complication of acute pulmonary embolism (PE). RVF-due to acute pressure overload is the leading cause of death in PE [1]. PE is also the third most common cause of cardiovascular death in the USA [2]. The absence of haemodynamic instability does not exclude the presence of right heart strain (RHS).

In this study, we have defined RHS in terms of echocardiographic findings- an example of some listed later in this introduction- to see if computed tomography pulmonary angiogram (CTPA) correlates with these findings. CTPA has taken over from ventilation-perfusion scanning and invasive pulmonary angiography as the gold standard for the radiological diagnosis of PE [3]. CTPA allows radiologists to accurately assess pulmonary vasculature to the subsegmental level [4]. A recent North American study reported a PE diagnosis rate on CTPA as low as 5%, reflecting a changing culture toward increased use and availability of CTPA. This altered approach to PE diagnosis reflects a significant change from nearly 50% positivity on testing for PE in the 1980s [5]. The provision and increased utilisation of these tests need to balance the benefits of diagnosing PE (or finding an alternative explanation for the patients’ symptoms) on CTPA with the risks of radiation exposure and the use of intravenous contrast.

Using transthoracic echocardiography (TTE) as the standard-bearer in diagnosing RHS, we aimed to assess how accurate CTPA and serum biomarkers are at predicting echocardiographic evidence of RHS. Signs of right heart strain on TTE include increased right: left ventricular ratio, abnormal septal motion, McConnell’s sign, tricuspid regurgitation, elevated pulmonary artery systolic pressure, decreased tricuspid annular plane systolic excursion (TAPSE), decreased S', pulmonary artery mid-systolic notching, 60/60 sign, and speckle tracking demonstrating decreased right ventricular free wall strain [6, 7].

Serum biomarkers of cardiac dysfunction and injury are often routinely checked in patients with confirmed or suspected PE awaiting confirmation. Troponin I is a protein subunit that inhibits muscle contraction in the absence of calcium. Primarily used as a marker to aid diagnosis of myocardial infarction, it is also useful in acute PE -when raised in this setting, it is believed to be an indicator of cardiac muscle damage and potentially signal RHS. Troponin is a recognised marker of short-term mortality in PE [8]. N-terminal prohormone brain natriuretic peptide (NT-pro-BNP) is a biomarker primarily measured in patients with suspected heart failure. It is released in greater quantities when cardiac muscle is stretched (most commonly in the setting of volume overload). Hence, it may be a possible marker of right heart strain in PE. Meta-analysis shows that echocardiographic RHS and elevated natriuretic peptides are useful short-term mortality markers, whereas the role of RHS on CTPA as a prognostic marker remains unclear [9]. Another meta-analysis suggests that an increased CTPA RV/LV ratio >1 is associated with a 2.5-fold increase in all-cause mortality and a 5-fold increase in PE-related mortality [10]. Other CTPA markers of right heart strain include septal deviation and inferior vena cava [11].

Our centre is a tertiary, university teaching hospital. As TTE isn’t readily available in all hospitals out of hours, we aimed to ascertain whether CTPA, troponin and NT-pro-BNP would provide a reliable and time-sensitive indication as to whether or not there may be co-existent cardiac injury as part of a patient's presentation with PE, as this in turn would have potential implications for prognosis and clinical management.

## 2 STUDY DESIGN AND METHODS

### 2.1 Patient Selection

We performed a retrospective chart review of 656 patients who had a CTPA over six monthsin our tertiary, university teaching hospital (July to December 2023). Patients’ data were collected and pseudonymized by tokenisation. We reviewed the patients’ clinical history, images, and the radiology reports of patients’ CTPAs, chest X-rays, and blood results, including D-dimer, troponin, and NT-pro-BNP. All patients aged 16 years of age or older who had a CTPA for an acute presentation of suspected PE within the above-specified dates were reviewed for possible inclusion in our study. Those excluded were under the age of 16 and those having a CTPA as a follow-up non-acute assessment. Basic patient demographics are reported in Table **1**.

### 2.2 Ethical Considerations

Ethical guidance and approval of project design and related considerations were provided by our institution’s affiliated hospital group. This study was entirely retrospective, and no patient interventions were performed by our group.

### 2.3 Radiology

CTPA scans were reported by consultant radiologists in our centre. Patients were then categorised into those who had an acute PE on CTPA and those who did not. Those who had a CTPA-confirmed PE were further divided into those with radiology-documented evidence of RHS and those without radiology-documented RHS. In our institution, the calling of right heart strain is usually as follows: currently, the primary means of identifying right heart strain on CTPA is through a reconstruction of the SAO projection to look at the right ventricle size and septum, looking for dilation of the ventricle and septal bowing/flattening. IVC reflux is not relied upon unless it is very marked. The majority of radiologists do not use the RV/LV ratio. The severity is not usually graded at our centre, but severe right heart strain is highlighted as it is more clinically relevant.

### 2.4 Laboratory Results

All blood results were taken from our institution's on-site haematology and biochemistry laboratories, using local reference ranges as documented below.

### 2.5 Echocardiography

All TTE studies were performed by trained cardiac technicians and reports finalised by consultant cardiologists, as is standard procedure in our institution. The Cardiologist interpretation of right heart function/strain in our institution is performed according to the European Society of Cardiology, British Society of Echocardiography, and American Society of Echocardiography guidelines. Specifically, right heart strain is diagnosed by an integrated assessment of right ventricular dilation (based on gender specific dimensions, indexed right ventricular end-diastolic area, and basal RV/LV ratio > 1), reduced right ventricular systolic function (TAPSE < 1.6 cm, tricuspid annulus S’ < 9.5 cm/sec, and RV fractional area change < 35%), interventricular septal flattening, TR velocity ≥ 2.8 m/sec, McConnell’s sign, and 60/60 sign [3, 7, 12, 13].

### 2.6 Statistics

Statistical analysis was performed using basic calculation methods and formulas to determine sensitivity, specificity, positive predictive value, and negative predictive value. Chi-square testing (using GraphPad PrismTM software) was used to determine the significance of the mortality data. Artificial intelligence was not used at any stage in this study.

## 3 RESULTS

### 3.1 CTPA

656 patients had a CTPA in our institution over the six-month period of our study. 128 (19.5%) patients had an acute PE diagnosed on CTPA. 31 (24.2%) of patients with PE had reported evidence of right heart strain on CTPA. Of those 31 patients, 25 also had a TTE. 16 (64%) of this patient group had evidence of acute RHS on their subsequent TTE. Of the 97 patients who had a PE with no radiological evidence of RHS, 39 had a TTE performed. 7 (17.9%) of these patients had evidence of RHS on TTE. This gives a sensitivity of 69.6% (95% confidence interval (CI) 49.1-84.4%), a specificity of 78% (95% CI 63.3-88.0%), a positive predictive value of 64% (95% CI 44.4-79.8%), and a negative predictive value of 82.1% (95% CI 67.3-91.0%) for CTPAs in predicting acute RHS. In total, 23/64 (35.9%) patients who had a TTE after a PE-positive CTPA had RHS (16/25 with RHS on CTPA, and 7/39 without RHS on CTPA).

### 3.2 NT-pro-BNP

26 patients with an acute PE on CTPA had both a TTE and an NT-pro-BNP sent in the acute setting. Using a cut-off value of 300ng/ml as per local reference range, NT-pro-BNP displayed a sensitivity of 92.9% (95% CI 79.4-100.0%), a specificity of 33.3% (95% CI 6.7-60.0%), a positive predictive value of 61.9% (95% CI 41.1-82.7%) and a negative predictive value of 80% (95% CI 44.9-100.0%) in predicting right heart strain in our patient cohort with PE.

### 3.3 Troponin

51 patients with an acute PE on CTPA had both a TTE and troponin sent in the acute setting. Using the local reference cut-off of 34ng/L, troponin demonstrated a sensitivity of 75% (95% CI 56.0-94.0%), a specificity of 58% (95% CI 40.7-75.4%), a positive predictive value of 53.6% (95% CI 35.1-72.0%) and a negative predictive value of 78.3% (95% CI 61.4-95.1%) in predicting right heart strain in patients with CTPA-proven PE.

If a higher troponin cut-off of 100ng/L (three times upper limit normal) was utilised, troponin then showed a sensitivity of 61.9% (95% CI 40.9-79.2%), a specificity of 80% (95% CI 62.7-90.5%), a positive predictive value of 68.4% (95% CI 46.0-84.6%) and a negative predictive value of 75% (95% CI 57.9-86.7%) in predicting RHS in our cohort. It should be noted that these analyses involving troponin, BNP, and CTPA had different numbers of patients, and a direct comparison cannot truly be made. These figures cannot be inferred to represent the full PE population.

When comparing the sensitivities of the various modalities, no statistically significant difference was noted in their ability to predict RHS in acute PE. The *P*-value of comparison between CTPA and BNP was 0.62, between CTPA and Troponin (34ng/L cut off) was 0.90, between Troponin (34ng/L cut off) and BNP was 0.77, and between Troponin (100ng/L cut off) and BNP was 0.78.

Of the remaining patients who had a CTPA with no evidence of an acute PE, 329/528 (62.23%) had another explanation for their presenting symptoms identified on their CT. Therefore, the overall diagnostic yield of CTPA was 69.7%. The radiological findings obtained on CT are detailed in Table **2**.

Overall, 118 (17.9%) of the total number of patients who had CTPAs were deceased six months after their CTPA. This included 5/31 (16.1%) of patients with right heart strain on CTPA and 19/97 (19.5%) of patients who had a PE but no CTPA evidence of RHS, reflecting a combined 18.8% mortality rate in those diagnosed with PE. 94/528 (17.8%) of patients who had no PE on their CTPA died during this period.

## 4 DISCUSSION

Acute pulmonary embolism with right heart strain is a life-threatening condition. Assessment of cardiac function is routinely performed as part of risk stratification following an acute PE. In this study, we aimed to ascertain the most reliable way to assess for RHS in the setting of acute PE. Many centres don’t have access to immediate out-of-hours echocardiography, so objective means of assessing severity of PE independent of TTE would potentially be of practical clinical utility. We have demonstrated that in our centre, CTPAs have a reasonable but not excellent sensitivity and specificity for diagnosing RHS. NT-pro-BNP has an excellent sensitivity but poor specificity. Using a troponin cut-off of three times the upper limit of normal maintains a reasonable sensitivity with a very good specificity for RHS in CTPA-diagnosed PE, provided an appropriate work-up has excluded acute coronary syndrome and other causes for an elevated troponin.

Prior research tells us that CT is highly sensitive, but not as specific as TTE for diagnosing RHS, hence why we use TTE as an accessible comparator in our work [14]. Other modalities such as cardiac MRI and right heart catheterization are more accurate but are not readily accessible in most centres and would be expensive, invasive, and burdensome on patients to perform in every PE case. International PE guidelines mentioned later in this discussion tell us that TTE is recommended for diagnosing RHS [3].

In a review comparing internationally recognised PE guidelines, the use of validated prognostic scores such as PESI was endorsed by the European Respiratory Society (ERS) and the European Society of Cardiology (ESC), as well as other organisations such as the American Society of Haematology (ASH). ESC/ERS and CHEST also recommend assessing right ventricular size and function to identify low-risk patients. The American Heart Association (AHA) and ASH recommend diagnosing patients as intermediate risk when RHS is detected, while ESC and ERS further divide this group into intermediate-low and intermediate-high risk categories based on combined imaging and biomarker abnormalities [15].

One of the major advantages of performing CTPA is that even when the CTPA shows that no PE is present, the detailed images of the lungs and related structures may afford an alternative explanation for dyspnoea, chest pain, or other symptoms that initially prompted the need for the scan. 2.3% of patients in our study had a likely new malignancy found, and 2.1% had acute rib, vertebral or sternal fractures. The counterargument that is often made is that the introduction of CTPA has led to overdiagnosis of PE, with rising incidence, minimal change in mortality, and lower-case fatality [16]. The proposed explanation is that more clinically insignificant emboli are being picked up through excessive CTPA use. However, it is noteworthy that available evidence has suggested that the risk of PE recurrence is similar in those with subsegmental PE and those with central PE [17].

One study reported that 45% of patients with acute PE will have RHS. In our study, it was approximately 24% on CTPA, rising to 35.9% on TTE [2]. Diagnosing the severity of PE is important in the initial clinical management and estimating outcomes in patients with acute PE. Patients diagnosed with any severity of PE will usually receive a minimum of three months of anticoagulation. The American College of Chest Physicians’ guidelines for venous thromboembolism give a weak, low-certainty recommendation that observation could be chosen over anticoagulation in patients with subsegmental PE, who are low risk for recurrence and have no evidence of proximal lower limb deep vein thrombosis. Patients with evidence of hypotension (*i.e.*, massive PE) who are not at a high bleeding risk should have systemically administered thrombolytic therapy [18]. The necessary major distinctions in initial therapy between different patient risk groups is why risk stratification is so important in acute PE.

Troponin is an established marker used in assessing PE. It has significance for predicting PE (50.8% *versus* 11.6% in those with PE compared with those without PE in one study, *p*<0.001); however, there was no statistical significance between TTE findings in those with PE with or without elevated troponin [19]. In prior studies, PE patients with elevated NT-pro-BNP or troponin were more likely to have adverse TTE findings than those without elevated biomarkers- as high as a twelve-fold increase in those with elevated NT-pro-BNP. The authors of that study suggest the possibility of not performing TTE in patients with normal biomarkers [20]. Our NT-pro-BNP findings mirror this finding, with 92.9% sensitivity observed; however, troponin was only 75% sensitive, and both performed poorly in terms of specificity and positive predictive value.

The 2019 ESC guidelines on management of acute PE recognise that right ventricular dysfunction may be present and affect early outcomes in patients with low clinical risk scores [3]. This adds additional importance to performing a thorough cardiac work-up in all patients with PE, regardless of clot burden on CTPA or severity of clinical presentation. This is highlighted in our study, whereby 17.9% of patients with no evidence of RHS on CTPA who went on to have a TTE had echocardiographic evidence of RHS. Our data reflects the recommendations of the above-mentioned 2019 guidelines, which also give a grade IIa recommendation to assess the right ventricle with echocardiography or biomarkers in all patients, regardless of PESI score.

Our study reports a mortality rate of 18.8% at 6 months in those diagnosed with PE, but interestingly there was still a 17.5% mortality rate at 6 months in those with no PE, with no statistical difference between the two groups (*p* = 0.78). This may reflect better understanding and advances in PE management, as well as the fact that significant proportions of patients referred for CTPA have malignancy, chronic respiratory disease, or other significant underlying conditions.

## 5 STUDY LIMITATION

A limitation of our study is that it is retrospective and single centre, and not all patients in the cohort were cared for or received dedicated input from the respiratory service. Also, this study is subject to some selection bias given that more unwell patients were more likely to get a TTE as an inpatient.

## INTERPRETATION/CONCLUSION

Our study demonstrates that while CTPA and biomarkers are useful and readily available indicators for cardiac involvement in PE, they lack the reliability and reassurance of quality provided by echocardiography. Our data suggest that patients who are diagnosed with an acute PE should be considered for TTE. Larger, multicentre, and prospective data are needed to truly assess the legitimacy of these diagnostic tools.

## Figures and Tables

**Table 1:** Demographics and testing for study patients.

**Basic Patient Characteristics (All Patients)**	-
Age	63.48 (16-92)
Male/female	48.3%/51.7%
**Patients diagnosed with PE work-up**	
NT-pro-BNP sent	34 (26.6%)
Troponin sent	83 (64.8%)
D-Dimer sent	94 (73.4%)
Chest X-ray performed	104 (81.25%)
TTE performed	64 (50%)

**Abbreviations:** PE = pulmonary embolism, NT-pro-BNP = NT-pro-BNP = N-terminal prohormone of brain natriuretic protein, TTE = Transthoracic echocardiogram

**Table 2:** Findings of CTPAs, detailing the likely cause of patients’ presenting symptoms.

**CTPA Findings**	-
Pulmonary embolism	128 (19.5%)
Infection	166 (25.3%)
*-Consolidation*	*119*
*-Mucous Plugging*	*35*
*-Atypical*	*12*
Unilateral pleural effusion	27 (4.1%)
Volume overload (Congestive cardiac failure/liver failure/renal failure, etc)	56 (8.5%)
Malignancy	31 (4.7%)
*-New*	*15*
*-Progression of known disease*	*16*
Pericardial effusion	8 (1.2%)
Pulmonary arterial hypertension	9 (1.4%)
Bony fracture	14 (2.1%)
Pneumothorax	5 (0.8%)
Interstitial lung disease	7 (1.1%)
*-New*	*3*
*-Progression of known disease*	*4*
Aortic dissection	1 (0.2%)
Haemothorax	1 (0.2%)
Vascular abnormality	1 (0.2%)
Sarcoidosis	1 (0.2%)
Pulmonary haemorrhage	1 (0.2%)
Liver abscess	1 (0.2%)

**Abbreviation:** CTPA = Computed tomography pulmonary angiogram

## Data Availability

All the data and supporting material are available within the article.

## LIST OF ABBREVIATIONS

RVF: Right ventricular failure
RHS: Right Heart Strain
CTPA: Computed Tomography Pulmonary Angiography
TAPSE: Tricuspid Annular Plane Systolic Excursion

## STANDARDS OF REPORTING

STROBE guidelines were followed.
